# Comparative analysis of instantaneous wave-free ratio and quantitative real-time myocardial contrast echocardiography for the assessment of myocardial perfusion

**DOI:** 10.3389/fcvm.2022.893647

**Published:** 2022-10-26

**Authors:** Li Liang, Yongxiang Zhu, Fangfang Li, Kai Guo, Shang Chang, Qian Li, Yaojun Zhang, Dongye Li

**Affiliations:** ^1^Department of Cardiology, Xuzhou Cancer Hospital, Xuzhou Medical University, Xuzhou, China; ^2^Department of Cardiology, Institute of Cardiovascular Diseases, Xuzhou Medical University, Xuzhou, China

**Keywords:** instantaneous wave-free ratio, RT-MCE, myocardial perfusion, coronary physiology, coronary microcirculation

## Abstract

**Background and hypothesis:**

The field of coronary artery physiology is developing rapidly and changing the practice of interventional cardiology. A new functional evaluation technique using the instantaneous wave-free ratio (iFR) has become an alternative to fractional flow reserve. Future research studies need to determine whether physiological indicators play a role in evaluating myocardial perfusion in the catheter room.

**Materials and methods:**

Thirty-eight patients scheduled for coronary angiography and iFR evaluation underwent a real-time myocardial contrast echocardiography (RT-MCE) examination at rest. The myocardial perfusion parameters (A, β, and A × β) on the myocardial perfusion curve were quantitatively analyzed using Q-Lab software. Coronary angiography and iFR assessment were completed within 1 week after the RT-MCE examination in all patients. Correlation analysis was used to identify iFR- and MCE-related indicators. The sensitivity and specificity of iFR in the quantitative detection of coronary microcirculation were obtained.

**Results:**

The correlation coefficients between iFR and A, β, and A × β were 0.81, 0.66, and 0.82, respectively. The cut-off value for iFR was 0.85 for microvascular ischemia detection, while the sensitivity and specificity for the diagnosis of myocardial perfusion were 90.7 and 89.9%, respectively. The receiver operating characteristic (ROC) curve area for iFR was 0.946 in the segments related to myocardial blood flow.

**Conclusion:**

The iFR is an effective tool for detecting myocardial microcirculation perfusion, with satisfactory diagnostic performance and a demonstrated role in physiological indices used for the perfusion assessment.

## Introduction

The principle of instantaneous wave-free ratio (iFR) is based on the theory that coronary microvascular resistance is constant during the diastolic wave-free period, which starts at 25% of diastolic duration and ends 5 ms before the end of the diastolic period. The ADVISE study has found that when the heart is in the diastolic wave-free period, the microvascular resistance in the coronary artery is the lowest and most stable, which is similar to the average resistance achieved during coronary hyperemia caused by adenosine and other vasodilators. The Pd/Pa measured during this period is a substitute for coronary flow during maximum hyperemia (1–6). Therefore, the cumulative data for iFR show that it is a reasonable alternative to fractional flow reserve (FFR) in a cardiac catheterization laboratory. Its advantage is that adenosine is not required (6–8). Real-time myocardial contrast echocardiography (RT-MCE) is a novel method for the evaluation of global and regional myocardial perfusion. Our preliminary study has suggested that quantitative RT-MCE indexes showed many advantages over qualitative indexes, as they are more reproducible and describe physiological features of both myocardial blood volume (MBV) and myocardial blood flow (MBF). Although MCE is a powerful tool for evaluating myocardial perfusion, it is still limited by time-consuming analysis and imaging artifacts (9).

Research in the field of coronary artery physiology is developing rapidly and changing the application of interventional cardiology (1–3, 10, 11). Many studies support the use of coronary physiological indicators in the cardiac catheterization room to evaluate epicardial stenosis. Understanding the differences between these indicators can provide the operators the flexibility to apply them in a clinical environment. However, only a few studies have determined whether physiological indexes played a role in evaluating myocardial microcirculation perfusion in the catheter room.

## Materials and methods

### Study population

A total of 38 consecutive patients with suspected coronary heart disease were enrolled in the study. All patients agreed to undergo MCE, coronary angiography (CAG), and iFR measurements. The inclusion criteria were age over 18 years, stable clinical condition, and normal regional and global left ventricular (LV) function in a resting state as determined by a routine echocardiography examination. The exclusion criteria included severe arrhythmia, atrial fibrillation, and contraindications for contrast media. All patients signed a written informed consent form. The study was in accordance with the Helsinki Declaration, and the protocol was approved by the hospital and regional medical ethics committees.

### Real-time myocardial contrast echocardiography image acquisition

Imaging was performed using Philips IE33 (Philips Medical Systems, Amsterdam, the Netherlands) equipped with an S5-1 sensor and low-power RT application. A commercially available second-generation contrast agent SonoVue (Bracco S. p. A, Milan, Italy) was used. A total of 59 mg of SonoVue were diluted in 5 mL of normal saline, and 2.5 mL of the solution was injected intravenously at an infusion rate of about 1 mL/min in each test. The injection parameters were adjusted for each patient according to the MCE images to ensure optimal myocardial enhancement. The process was operated by a volumetric infusion pump through a 20G vial in the vein of the proximal forearm. The SonoVue infusion rate was carefully adjusted to minimize far-field attenuation and optimize myocardial opacity.

In the study setup, the best balance between myocardial contrast enhancement and attenuation was achieved at a very low mechanical index (0.1). The color gain was adjusted to reduce the signal-to-noise to minimize the noise in the myocardium and LV cavity. Furthermore, the time gain compensation was adjusted to obtain uniform signal intensity and reduce the noise from the myocardium, pericardium, mitral valve, and epicardium. All settings were initially optimized and remained unchanged in each separate acquisition (12–14).

The long-axis and apical two- and four-chamber section readings were obtained by an experienced ultrasound doctor. Once the cavity and myocardial contrast medium turbidity reached a stable state, microbubble destruction was performed with a short burst (flash) of eight frames with a high mechanical index (1.6). The mechanical index was automatically readjusted to the low level immediately after the flash so that microbubbles refilling could be continuously observed in the next 10 cardiac cycles. For each view, the program was repeated at least two times to obtain 15 cardiac cycles and store them as raw data.

### Real-time myocardial contrast echocardiography data analysis

**FIGURE 1:**
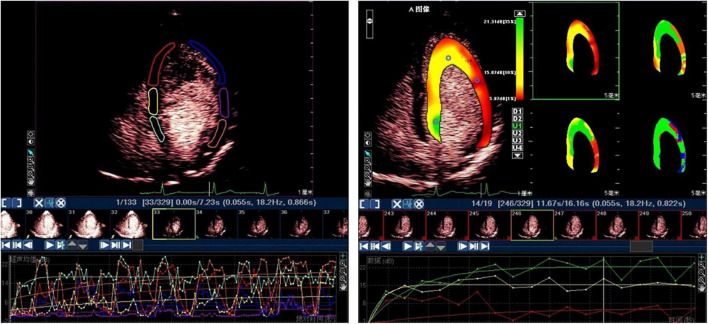
The subsequent myocardial refilling curve by ROI method **(left)** and by parameter display method **(right)**.

The MCE sequence was transmitted to a personal computer for offline analysis. The quantitative analysis tool in Q-Lab 4.2 software (Philips Medical Systems, Amsterdam, the Netherlands) was used to analyze the images using a random blind method. The data were automatically measured in the region of interest (ROI) and manually positioned using a standard 17-segment LV model. The ROI at the end of each contraction frame after the first flash was automatically copied to all subsequent selected frames. These ROIs were manually realigned frame by frame. According to the functional relationship between indicators, the segmental MCE parameter A was expressed as the average MBV, β represented the average MBF speed, and A × β showed the average MBF (Figure 1) (15–19).

Using repeated blind analysis of 10 randomly selected patients, A and β intra- and inter-observer variability values for all parameters were obtained after at least 8 weeks.

### Coronary angiography and instantaneous wave-free ratio

All patients underwent a multi-view selective coronary angiography examination within 1 week after RT-MCE. Coronary angiography was analyzed by an independent experienced observer who was unaware of RT-MCE data. The guiding catheter was used after a coronary angiography evaluation and the iFR values for LAD, LCX, and RCA were measured. All procedures were performed according to the guidelines for iFR measurements. The sensor catheter was placed at least 3 cm below the farthest lesion in the coronary artery. If the distance was <3 cm or there was no stenosis, the catheter was placed at the distal end of the coronary artery as far as possible to avoid sticking to the wall and missing potential lesions. Intracoronary nitroglycerin (100 ug) was administered before the measurement. The iFR value was automatically calculated online using proprietary software (version 3.3.0, Volcano Harvest, Volcano Corporation, Rancho Cordova, CA, USA).

### Statistical analysis

Continuous variables were expressed as the mean ± standard deviation (SD). The MCE parameters from two tests were averaged to minimize the impact of analysis error before performing statistical analysis. Correlation analysis was used to evaluate iFR- and MCE-related indicators. *P* < 0.05 was defined as statistically significant. The predictive ability of iFR was calculated using the ROC curve. The Classification Tree was used for the multifactorial analysis of myocardial ischemia identified by MCE. Intra- and inter-observer variability values were calculated as SD of the mean difference and expressed as a percentage of the mean.

## Results

### Patient characteristics and coronary angiography

Forty patients were referred for a diagnostic coronary angiography examination due to suspected coronary heart disease. In addition, two patients were excluded due to insufficient image quality of all standard apical views (not all coronary artery regions were fully visualized), leaving a total of 38 patients for comparative quantitative analysis. Coronary angiography detected a total of 74 coronary artery stenosis (LM, 2; LAD, 25; D_1_, 11; LCX, 13; OM, 9; and RCA, 14). Patient baseline data and coronary artery disease (CAD) distribution characteristics are summarized in Table 1.

**TABLE 1:** Patient characteristics and coronary angiography.

	*n*	Min	Max	Mean
Gender	27, male			–
Age	38	43	82	59.92 ± 10.66
Hypertension	25	–	–	–
Diabetes	4	–	–	–
Obesity	11	–	–	–
Smoking history	20	–	–	–
Drinking history	9	–	–	–
LM, %	2	60	60	60.00
LAD, %	25	20	100	70.44 ± 28.93
D_1_, %	11	20	99	46.73 ± 25.71
OM, %	9	20	95	43.89 ± 30.19
LCX, %	13	30	100	72.69 ± 23.95
RCA, %	14	20	100	70.71 ± 28.34
CHOL (mmol/L)	38	2.85	8.22	5.08 ± 1.10
HDL (mmol/L)	35	0.55	2.35	1.25 ± 0.36
Tn I (ng/ml)	2	0.89	0.91	0.90 ± 0.01

LM, left main; LAD, left anterior descending; D_1_, first diagonal; LCX, left circumflex; OM, obtuse marginal; RCA, right coronary artery; HDL, high-density lipoprotein.

### Real-time myocardial contrast echocardiography data

A total of 561 myocardial segments were obtained from 608 myocardial segments in 38 patients. Due to poor image quality, 47 segments were excluded. The numbers of myocardial segments represented by LAD, LCX, and RCA were 214, 175, and 172, respectively. There were 204 basal, 214 middle, and 143 apical myocardial segments. The intra- and inter-observer coefficients of variation based on quantitative analysis were 93.8 and 94.6%, respectively.

### Quantitative myocardial contrast echocardiography and instantaneous wave-free ratio data

**FIGURE 2:**
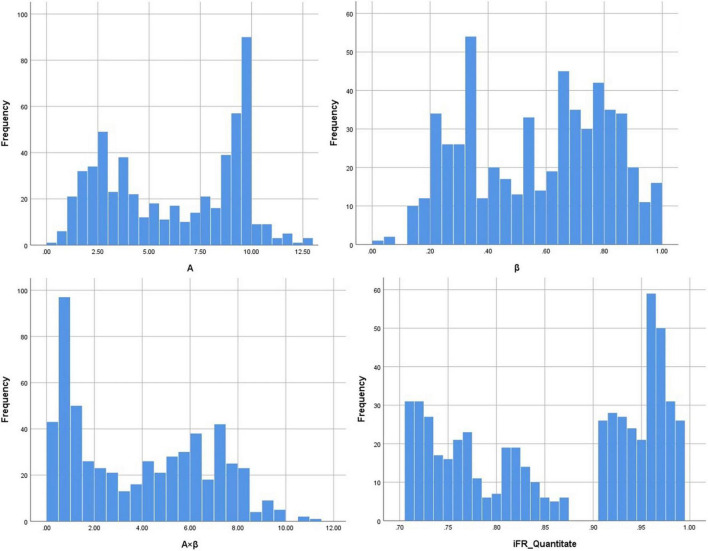
The data distribution of A (myocardial blood volume), β (myocardial blood flow velocity), A × β (myocardial blood flow), and iFR (instantaneous wave-free ratio).

A total of 271 ischemic segments were detected out of 561 total evaluated segments using quantitative analysis. The values for A, β, and A × β in 561 segments were 6.17 ± 3.22, 0.57 ± 0.24, and 3.90 ± 3.86, respectively. Then, 207 ischemic segments were detected by the quantitative analysis of coronary angiography results. The corresponding iFR values for 561 effective segments in the myocardium were obtained based on the 16-segment method, with an iFR value of 0.87 ± 0.10. Quantitative MCE and iFR data are shown in Figure 2.

### Correlation analysis between instantaneous wave-free ratio and myocardial contrast echocardiography

**FIGURE 3:**
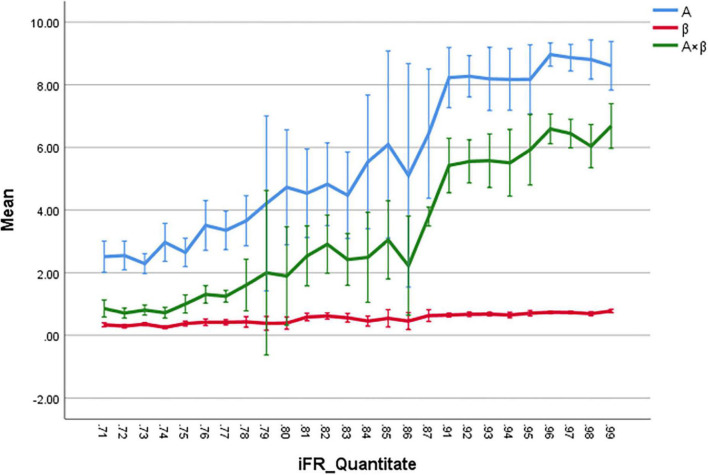
The correlation coefficients between iFR and A, β, and A × β are 0.81, 0.66, and 0.82, respectively (*P* < 0.01). The iFR and A, and A × β are highly correlated, respectively (*P* < 0.05).

**FIGURE 4:**
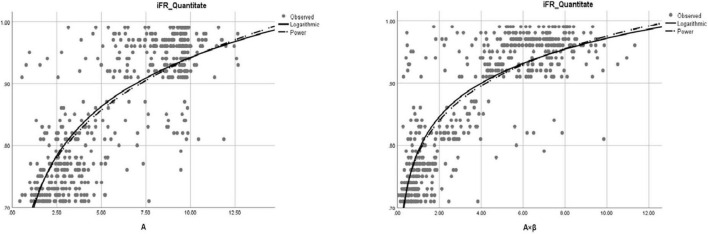
The fitting of logarithmic and power curves between iFR and A (myocardial blood volume) and A × β (myocardial blood flow).

The correlation coefficients between iFR and A, β, and A × β were 0.81, 0.66, and 0.82, respectively (*P* < 0.01). According to these data, there was a significant correlation between iFR and A as well as A × β (*P* < 0.05; Figure 3). The logarithmic and power curve fitting between iFR and A as well as A × β was ideal (*P* < 0.05; Figure 4).

### Instantaneous wave-free ratio data at myocardial segment perfusion level derived using quantitative myocardial contrast echocardiography

**FIGURE 5:**
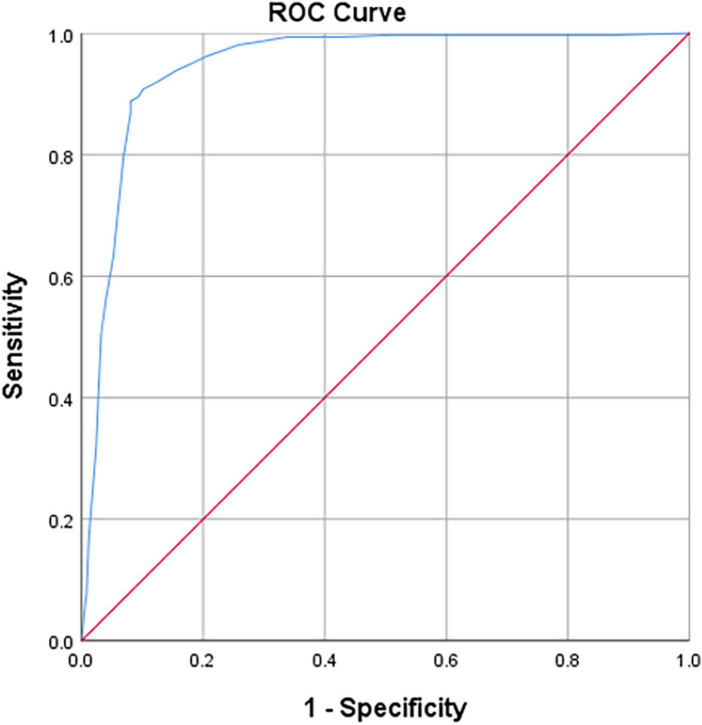
Receiver operator characteristic (ROC) curves on iFR vs. MCE (myocardial blood flow, MBF) results (*P* < 0.01). Diagonal segments are produced by ties.

The A × β < 2.75 (20, 21) is the gold standard for myocardial ischemia, with the cut-off for iFR of 0.85 for the diagnosis of coronary microcirculation. The sensitivity and specificity values for iFR-based detection of myocardial ischemia were 90.7 and 89.9%, respectively. The ROC curve area for iFR was 0.946 for the segments related to myocardial blood flow (*P* < 0.01; Figure 5).

### Classification of instantaneous wave-free ratio and myocardial contrast echocardiography (A and A × β)

**FIGURE 6:**
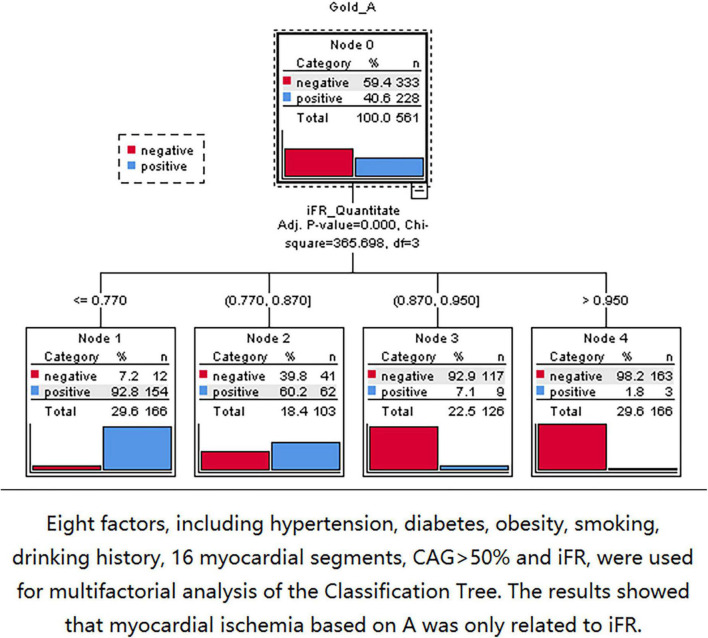
The 0.77, 0.87, and 0.95 nodes can be found by using classification tree analysis and A < 4.58 as the cut-off point of myocardial blood volume reduction.

**FIGURE 7:**
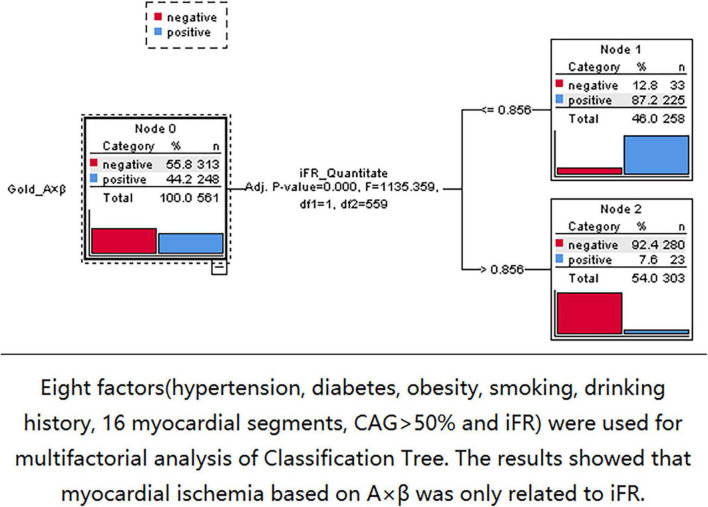
The 0.856 node can be found by using classification tree analysis and A × β < 2.75 as the cut-off point of myocardial blood flow reduction.

Three nodes were identified using Classification Tree analysis, with A < 4.58 serving as the cut-off point for myocardial blood volume reduction. The three nodes were 0.77, 0.87, and 0.95, respectively (Figure 6). One node (0.856) was determined using Classification Tree analysis and A × β < 2.75 as the cut-off point for myocardial blood flow reduction (Figure 7).

## Discussion

At present, functional evaluation of coronary artery disease using FFR is a routine practice in percutaneous coronary intervention and is officially recommended by European guidelines (1). In recent years, a new functional evaluation technique employing iFR has become an alternative to FFR. Many previous studies have shown that iFR demonstrates encouraging diagnostic accuracy that is highly consistent with the FFR results (22–29). Although many large-scale clinical trials have verified the application value of iFR, the association between iFR and myocardial ischemia based on MCE evaluation remains unexplored (23, 30–33).

Furthermore, the iFR measurement requires the use of pressure lines, but adenosine is not required. Therefore, the iFR assesses coronary pressure rather than MBF and coronary artery ischemia rather than myocardial ischemia. Myocardial blood flow and myocardial ischemia are the causes of coronary atherosclerotic heart disease. RT-MCE is a reliable method for evaluating regional myocardial perfusion. It has been reported that MBF measurements obtained by intermittent and quantitative RT-MCE techniques are well correlated with Doppler blood flow measurements in animals and humans (20, 21). In the present study, the correlation between iFR and MCE was compared from the perspective of myocardial perfusion. The study tried to explore the diagnostic value and application of iFR in evaluating myocardial blood flow perfusion and to expand the decision-making value of iFR in interventional therapy.

The main findings of the present study revealed a good correlation between iFR and multiple indicators of myocardial perfusion. In particular, iFR was highly correlated with regional MBV (A) and MBF (A × β), which was expected. There was also a very good curve fitting between iFR and A as well as A × β. However, the correlation between iFR and regional MBF velocity (β) was relatively low. The coronary artery is characterized by high blood flow. Blood volume and blood flow velocity are two factors that determine blood flow and are mutually restricted both in the epicardial artery and microcirculation (34). This may be related to the speed index itself serving as a secondary indicator.

Because the coronary artery system consists of three components with different functions (conducting epicardial coronary arteries, arterioles, and capillaries), myocardial ischemia may occur when any of these components fail. However, macrovascular and microvascular diseases are characterized by different processes and have different susceptibility factors (35). In the present study, there were only 23 (4.1%) myocardial segments with iFR > 0.856 and A × β < 2.75. Thus, there was no primary microvascular ischemia. Furthermore, the study tried to explore the factors related to myocardial ischemia based on MCE. Eight factors, including hypertension, diabetes, obesity, smoking history, drinking history, 16 myocardial segments, CAG > 50%, and iFR, were considered. The Classification Tree analysis results showed that the myocardial ischemia based on MCE was only associated with iFR and was not associated with CAG > 50%.

In our previous studies, MCE has been demonstrated to be a better method for assessing myocardial perfusion than radionuclide tests, such as SPECT (20). The myocardial ischemia diagnosis cut-off values for A, β, and A × β were 4.58, 0.64, and 2.73, respectively. Using these cut-off points as the gold standard, the iFR was demonstrated to have a very good diagnostic value for evaluating myocardial ischemia. Most of the experiments in the previous studies have focused on the comparison between iFR and FFR. The present study was the first to identify the cut-off point for iFR to diagnose myocardial ischemia at the myocardial perfusion level based on MCE.

The present study considered both the MBV and MBF, as well as the possible omission of positive patients and the reduction of unnecessary medical expenditure. It was believed that the gray area of iFR existed, as J. M. Lee and E. M. Baile’s research in previous studies (27–31). As described in the above results, interventional therapy should be considered when the iFR value is between 0.85 and 0.95. Myocardial ischemia could only be demonstrated for iFR < 0.85. For patients with iFR values ranging from 0.85 to 0.95, FFR should be considered for further assessment of coronary artery function to perform an objective evaluation of interventional treatment decisions. Determining the application value of iFR at the myocardial perfusion level was the most significant aim of the present study. MCE had a high temporal and spatial resolution and was highly sensitive to the diagnosis of myocardial ischemia. A significant correlation between iFR and MCE was thus identified, which will provide a good reference for guiding interventional therapy using iFR.

### Study limitations

The present study compared the correlation between myocardial perfusion indices derived from MCE and iFR. However, real cases of coronary artery lesions are much more complicated. For example, multiple lesions, microangiopathy, and collateral circulation compensation all affect the results. Subsequent subgroup analysis should be performed in future.

## Conclusion

The iFR was an effective method for evaluating myocardial perfusion. The iFR demonstrated a reasonable diagnostic performance and a role in physiological indexes for evaluating coronary microcirculation. To effectively improve its clinical applicability, stronger comparative detection techniques, and simplified data analysis and display are still needed. Furthermore, different coronary artery physiology techniques need to be compared in larger controlled studies.

## Data availability statement

The original contributions presented in this study are included in the article/supplementary materials, further inquiries can be directed to the corresponding authors.

## Ethics statement

The studies involving human participants were reviewed and approved by the Medical Research Ethics Committee of Xuzhou Cancer Hospital. The patients/participants provided their written informed consent to participate in this study. Written informed consent was obtained from the individual(s) for the publication of any potentially identifiable images or data included in this manuscript.

## Author contributions

LL, YJZ, DL, YZ, FL, SC, and QL: concept. LL, YZ, FL, KG, and SC: design. DL, YJZ, LL, QL, SC, and KG: supervision. YJZ, DL, and LL: fundings and writing. YZ, FL, KG, SC, QL, and LL: materials. FL, YZ, QL, and SC: data collection or processing. KG, SC, and QL: analysis or interpretation. LL, YJZ, DL, YZ, and FL: literature search. LL, YJZ, DL, QL, SC, YZ, FL, and KG: critical review. All authors contributed to the article and approved the submitted version.
